# Supplementary material for: Stacking Model‐Based Classifiers for Dealing With Multiple Sets of Noisy Labels
Source: Biom J. 2025 Mar 12;67(2):e70042. doi: 10.1002/bimj.70042 (PMC11898607; doi:10.1002/bimj.70042)
Supplement: Supplementary file 1 — Supporting Information [file BIMJ-67-e70042-s001.zip › density-based-ensemble-model-main/competitors_real_data/netlab-master/netlab-master/help/gtminit.htm]

Netlab Reference Manual gtminit


# gtminit

## Purpose

Initialise the weights and latent sample in a GTM.

## Synopsis

```
net = gtminit(net, options, data, samptype)
net = gtminit(net, options, data, samptype, lsampsize, rbfsampsize)
```

## Description

`net = gtminit(net, options, data, samptype)` takes a GTM `net`
and generates a sample of latent data points and sets the centres (and
widths if appropriate) of
`net.rbfnet`.

If the `samptype` is `'regular'`, then regular grids of latent
data points and RBF centres are created. The dimension of the latent data
space must be
1 or 2. For one-dimensional latent space, the `lsampsize` parameter
gives the number of latent points and the `rbfsampsize` parameter
gives the number of RBF centres. For a two-dimensional latent space,
these parameters must be vectors of length 2 with the number of points
in each of the x and y directions to create a rectangular grid. The
widths of the RBF basis functions are set by a call to `rbfsetfw`
passing `options(7)` as the scaling parameter.

If the `samptype` is `'uniform'` or `'gaussian'` then the
latent data is found by sampling from a uniform or
Gaussian distribution correspondingly. The RBF basis function parameters
are set
by a call to `rbfsetbf` with the `data` parameter
as dataset and the `options` vector.

Finally, the output layer weights of the RBF are initialised by
mapping the mean of the latent variable to the mean of the target variable,
and the L-dimensional latent variale variance to the variance of the
targets along the first L principal components.

## See Also

`gtm`, `gtmem`, `pca`, `rbfsetbf`, `rbfsetfw`

---

**Pages:**
Index


---

Copyright (c) Ian T Nabney (1996-9)
